# Multi-factors including Inflammatory/Immune, Hormones, Tumor-related Proteins and Nutrition associated with Chronic Prostatitis NIH IIIa+b and IV based on *FAMHES* project

**DOI:** 10.1038/s41598-017-09751-8

**Published:** 2017-08-22

**Authors:** Yang Chen, Jie Li, Yanling Hu, Haiying Zhang, Xiaobo Yang, Yonghua Jiang, Ziting Yao, Yinchun Chen, Yong Gao, Aihua Tan, Ming Liao, Zhen Lu, Chunlei Wu, Xiaoyin Xian, Suchun Wei, Zhifu Zhang, Wei Chen, Gong-Hong Wei, Qiuyan Wang, Zengnan Mo

**Affiliations:** 1 Institute of Urology and Nephrology, The First Affiliated Hospital of Guangxi Medical University, Nanning, China; 2 Center for Genomic and Personalized Medicine, Guangxi Medical University, Nanning, Guangxi Zhuang Autonomous Region China; 3 Guangxi key laboratory for genomic and personalized medicine, Nanning, Guangxi Zhuang Autonomous Region China; 4 Guangxi collaborative innovation center for genomic and personalized medicine, Nanning, Guangxi Zhuang Autonomous Region China; 5 Guangxi key laboratory of colleges and universities, Nanning, Guangxi Zhuang Autonomous Region China; 6 Biocenter Oulu, University of Oulu, Oulu, Finland; 7 Faculty of Biochemistry and Molecular Medicine, University of Oulu, Oulu, Finland; 8 The Guangxi Zhuang Autonomous Region Family Planning Research Center, Nanning, Guangxi China

## Abstract

Chronic prostatitis (CP) is a complex disease. Fragmentary evidence suggests that factors such as infection and autoimmunity might be associated with CP. To further elucidate potential risk factors, the current study utilized the Fangchenggang Area Male Health and Examination Survey (*FAMHES*) project; where 22 inflammatory/immune markers, hormone markers, tumor-related proteins, and nutrition-related variables were investigated. We also performed baseline, regression, discriminant, and receiver operating characteristic (ROC) analyses. According to NIH-Chronic Prostatitis Symptom Index (NIH-CPSI), participants were divided into chronic prostatitis/chronic pelvic pain syndrome (CP/CPPS, pain ≥ 4; divided into IIIa and IIIb sub-groups) and non-CPPS (pain = 0; divided into IV and normal sub-groups). Analyses revealed osteocalcin as a consistent protective factor for CP/CPPS, NIH-IIIb, and NIH-IV prostatitis. Further discriminant analysis revealed that ferritin (*p* = 0.002) and prostate-specific antigen (PSA) (*p* = 0.010) were significantly associated with NIH-IIIa and NIH-IV prostatitis, respectively. Moreover, ROC analysis suggested that ferritin was the most valuable independent predictor of NIH-IIIa prostatitis (AUC = 0.639, 95% CI = 0.534–0.745, *p* = 0.006). Together, our study revealed inflammatory/immune markers [immunoglobulin E, Complement (C3, C4), C-reactive protein, anti-streptolysin, and rheumatoid factors], hormone markers (osteocalcin, testosterone, follicle-stimulating hormone, and insulin), tumor-related proteins (carcinoembryonic and PSA), and a nutrition-related variable (ferritin) were significantly associated with CP or one of its subtypes.

## Introduction

Prostatitis is a common male urogenital disease, with high heterogeneity and unclear pathogenesis. In order to define the disease, in 1999, the National Institute of Diabetes and Digestive and Kidney Diseases (NIDDK) devised a new classification system^1^, in which four types were specified: acute prostatitis (NIH-I), chronic bacterial prostatitis (NIH-II), chronic prostatitis/chronic pelvic pain syndrome (CP/CPPS, NIH-III), and asymptomatic inflammatory prostatitis (NIH-IV). NIH-III and IV are considered as nonbacterial types of prostatitis. Among nonbacterial prostatitis, CP/CPPS (NIH-III) is characterized by pelvic or perineal pain without evidence of urinary tract infection^2^, with accompanying symptoms such as dysuresia, arthralgia, depression, and hysteria. Recent studies suggest that CP/CPPS is associated with many diseases, including diabetes^3^, prospermia^4^, irritable bowel syndrome^5^, and male reproductive dysfunction^6^. In contrast, NIH-IV prostatitis is asymptomatic, and only evident with the presence of inflammatory cells in expressed prostatic secretion, or histological prostate biopsy specimens.

Although nonbacterial prostatitis is not a fatal disease, one study showed that this was a problem of social cost, and had similar effects on quality of life as that on the patients with diabetes mellitus and myocardial infarction^7^. Furthermore, several studies indicated that it might also lead to the development of prostate cancer^8, 9^, whereas this was still controversial^10^. There are estimated five million new prostatitis cases diagnosed annually, with 2.2–9.7% prevalence worldwide^11, 12^. In 2009, Liang *et al*.^13^ conducted a population-based cross-sectional survey in China; where 15 000 men, aged between 15 and 60 years, were surveyed. The results showed that 8.4% of participants had prostatitis-like symptoms, and 5.4% were diagnosed with CP according to the National Institute of Health-Chronic Prostatitis Symptom Index (NIH-CPSI) criteria, with a cost of 8059 China Yuan (CNY) annually for each patient. However, to date, no effective therapeutic approaches have been proposed^14^.

In order to treat the enormous numbers of nonbacterial prostatitis patients (CP/CPPS and NIH-IV prostatitis), recent studies have attempted to understand the possible pathogenesis of the condition. Similar to bacterial prostatitis, infection-related factors, including bacteria, viruses, and yeast, were first under suspicion as potential causative factors for CP/CPPS (NIH-III)^15–18^. In 2011, Rudick *et al*.^19^ induced sustained chronic pelvic pain in mice with Uropathogenic Escherichia coli, and confirmed the role of bacteria in the development of CP/CPPS. However, another case-control study revealed no difference in the rate of positive bacteria cultures in prostatic secretion between CP/CPPS and asymptomatic men^20^. Therefore, infections appear not to be the single causative factor. Additionally, the episodic and relapsing symptoms, and the discovery of seminal plasma antigens in CP/CPPS, suggest that autoimmune components might contribute to symptoms in some men with prostatitis^21^. Moreover, prostate specific antigen (PSA) was identified to be a potential candidate antigen for further targeted therapy in CP/CPPS^22^. In addition, hormones and psychology might also contribute to potential etiologies^23, 24^. Further, a wide range of self-reported medical conditions have been suggested to play a key role CP/CPPS pathogenesis^25^. We have previously reported that age, smoking, alcohol consumption, and lower levels of education might be risk factors for NIH-IV prostatitis^26^. While the accumulating evidence point out diverse risk factors, few studies have been conducted by focusing on comprehensive biochemical markers, including inflammatory/immune markers, hormone markers, tumor-related proteins, and nutrition markers.

Here we present thus far the most comprehensive study, which is based on the Fangchenggang Area Male Health and Examination Survey (*FAMHES*) project in Guangxi province, China. This project was primarily conducted in the Medical Center in Fangchenggang First People’s Hospital. All participants were asked to complete a comprehensive questionnaire, which included the NIH-CPSI. Blood samples were also tested for 22 specific biochemical markers, covering the common biochemical items tested in the hospital. These 22 markers include inflammatory/immune markers, hormone markers, tumor-related proteins, and nutrition components, which could reveal the potential pathogenic factors and biomarkers involved in CP/CPPS and NIH-IV prostatitis.

## Methods and Materials

### Brief overview of participants

As described in our previous studies^27, 28^, the FAMHES project was carried out from September to December 2009, and involved a routine physical examination at the Medical Center in Fangchenggang First People’s Hospital. The sample comprised 4303 non-institutionalized Chinese men aged 17 to 88 years old, who were from the Fangchenggang area of Guangxi, China. Following this, 3593 participants were followed via interviews, and asked to complete a comprehensive demographic and health survey. The response rate was 83.5%. Before analysis, we removed juvenile subjects to ensure that only data from adult participants (aged ≥ 18 years old) were included. Written informed consent was obtained, and the study was approved by the medical ethics committee of Guangxi Medical University. All the experiments were performed in accordance with relevant guidelines and regulations of this medical ethics committee.

### Selection of biochemical markers and serum measurements

Blood samples were collected from all the participants, and 22 specific biochemical markers were tested; these covered the common markers tested in the hospital, including inflammatory/immune markers, hormone markers, tumor-related proteins, and nutrition components. Specifically, the markers were: (I) inflammatory/immune markers: complement 3 (C3), complement 4 (C4), immunoglobulin E (IgE), immunoglobulin A (IgA), immunoglobulin G (IgG), immunoglobulin M (IgM), C reactive protein (CRP), antistrptolysin o (ASO), and rheumatoid factor (RF); (II) hormone markers: estradiol (E2), folic acid (FOL), follicle-stimulating hormone (FSH), luteinizing hormone (LH), osteocalcin (Osteoc), sex hormone binding globulin (SHBG), testosterone (TESTO), and insulin; (III) tumor-related proteins: alpha-fetoprotein (AFP), carcino embryonie antigen (CEA), and prostate specific antigen (PSA); and (IV) nutrition markers: vitamin B12 (B12) and ferritin (FERR).

The laboratory markers were measured in the testing center of the Department of Clinical Laboratory at the First Affiliated Hospital of Guangxi Medical University in Nanning, China. Blood samples were collected from participants between 8:00–11:00 a.m., after fasting for at least 8 h (overnight). The blood samples were centrifuged within 15–25 min, and stored at −80 °C. Further details are available in our previous reports^27–29^.

### Sample screening and grouping

Strict exclusion criteria were applied to ensure the samples from eligible participants. These criteria included: (I) incomplete individual participant information; (II) incomplete NIH-CPSI score data, especially for the pain assessment section; (III) incomplete data for any of the biochemical markers investigated in this study, or refused to provide the blood samples; (IV) participants with hypertension, myocardial infarction, congestive heart failure, stroke, hyperthyroidism, rheumatoid arthritis, acquired immune deficiency syndrome, any kind of cancer, or with a history of trauma or surgery, which might influence the levels of inflammatory/immune markers, hormones, tumor-related proteins, or nutrition markers; (V) currently taking drugs which could influence the status of biochemical markers being investigated, such as vitamins, antidiabetic medicines, non-steroidal anti-inflammatory drugs, antibiotics, cimetidine, glucocorticoids, or other steroidal drugs. After application of the exclusion criteria, 1682 participants were selected.

CP/CPPS and NIH-IV prostatitis were defined according to NIH-CPSI. There are nine questions in this scoring system, with a total score of 43. These questions are classified into three parts: evaluation of pain, urinary symptoms, and impact on quality of life. In 2001, Nickel *et al*.^30^ recommended pain = 4 as the threshold to distinguish prostatitis-like symptoms from none. Based on this standard, CP/CPPS in our study was identified as a score of pain ≥ 4. For non-CPPS, a pain score of 0 was used to eliminate possible controversies among the men scoring 1–3.

In addition, the participants in our FAMHES project were also encouraged to undergo a digital rectal examination for the collection of an expressed prostatic secretion (EPS) specimen^31^. In total, 1779 samples were successfully acquired. All EPSs were sent immediately to the clinic laboratory. For each sample, at least 25 fields were examined, with a high power microscope (HP; 400X). Leukocytes were counted and divided to determine the mean value per HP. The degree of inflammation in each EPS specimen was classified into five groups: (I) occasional or few: 0–9 leukocytes/HP; (II) 1+: 10–20 leukocytes/HP; (III) 2+: 21–30 leukocytes/HP; (IV) 3+: 31–40 leukocytes/HP; (V) 4+: 40 leukocytes/HP. Next, according to the threshold of 10 leukocytes/HP (inflammation group ≥ 10/HP; non-inflammation group < 10/HP), the NIH-IIIa, NIH-IIIb, NIH-IV or normal sub-types were subdivided from the CP/CPPS and non-CPPS groups^32^.

### Essential characteristics and physical examination

Information was collected from participants face-to-face interviewed by experienced personnel, using a standardized protocol. During this process, a comprehensive survey was completed. Data on essential characteristics such as age, sex, family address, jobs, smoking, alcohol consumption, etc. were collected, and physical examinations (height, weight, waistline, hipline, etc.) were performed. With regard to the essential characteristics, smoking status was divided into two categories: never a smoker or currently a smoker. Alcohol consumption was defined as the consumption of alcohol, including beer, wine, and hard liquor, within the participant’s lifetime. Waist circumference was measured at the midpoint between the inferior costal margin and the superior iliac crest on the mid-axillary line. The hipline was the maximum circumference over the buttocks. From these, the waist-hip ratio (WHR) was calculated. Additionally, body mass index (BMI) was acquired by measuring body weight with thin clothing and height without shoes. In this study, five variables (age, smoking, alcohol consumption, BMI, and WHR) were applied as covariates in all analyses.

### Statistical analysis

Before analysis, the distributions of the continuous variables were tested with the Shapiro-Wilks test. The biochemical markers that did not satisfy the Gaussian distribution assumption were transformed logarithmically to be relatively normal. Of the 1682 eligible participants, 1439 were included in the study based on the strict exclusion criteria; 198 of these participants were classified as CP/CPPS (pain ≥ 4) and 1241 were classified as non-CPPS (pain = 0), after excluding 243 participants with a pain score of 1–3. Further, among the 1439 samples, only 676 had results for EPS. According to the inflammation markers in the EPS, 35 participants were classified as NIH-IIIa, 86 were classified as NIH-IIIb, 139 were classified as NIH-IV prostatitis, and 416 were classified as normal. In order to investigate the association between pain and the included biochemical markers, Pearson correlation analyses were performed initially in three groups (CP/CPPS vs non-CPPS, IIIa vs normal, and IIIb vs normal). Next, baseline analyses were conducted in CP/CPPS vs non-CPPS, IIIa vs normal, IIIb vs normal, and IV vs normal groups, using the Student’s t-test, Mann-Whitney U test, and the Χ^2^ test, where appropriate. Additionally, both linear regression and binary logistic regression analyses were performed, in which four models (unadjusted, age-adjusted, age and smoking-adjusted, and multivariate-adjusted) were investigated. In the multivariate-adjusted model, the covariates were age, smoking, alcohol consumption, BMI, and WHR. To confirm the results obtained from these analyses, and to potentially uncover more interesting results, all biochemical markers were quartered (Q1 < 25%; 25% ≤ Q2 ≤ 50%; 50% < Q3 ≤ 75%; Q4 > 75%). The lowest level of every biochemical marker (Q1 < 25%) was treated as the reference for multinomial logistic regression analysis. In these analyses, four models were investigated [unadjusted (Model 1); age-adjusted (Model 2); age and smoking-adjusted (Model 3); multivariate-adjusted (Model 4)]. Moreover, in order to evaluate the predictive functions of these identified models for CP/CPPS and NIH-IV prostatitis, Receiver Operating Characteristic (ROC) analyses were also performed by adding the variables and covariates that were statistically associated with CP/CPSS and NIH-IV to a multivariable-adjusted logistic regression model^33^. Finally, discriminant analysis and principal component analysis (PCA) was performed. Associations were primarily assessed by the odd ratio (OR), 95% confidence interval (CI), and *p* value. An OR > 1 indicated that the biochemical marker was a risk factor for prostatitis; in contrast, an OR < 1 indicated that the biochemical marker was a protective factor. All the analyses were conducted with SPSS version 16.0 software (SPSS Inc., Chicago, IL, USA) and R. The statistical tests were two-tailed.

## Results

### Pain in CP/CPPS associated with inflammatory/immune factors and tumor-related proteins

**Figure 1:**
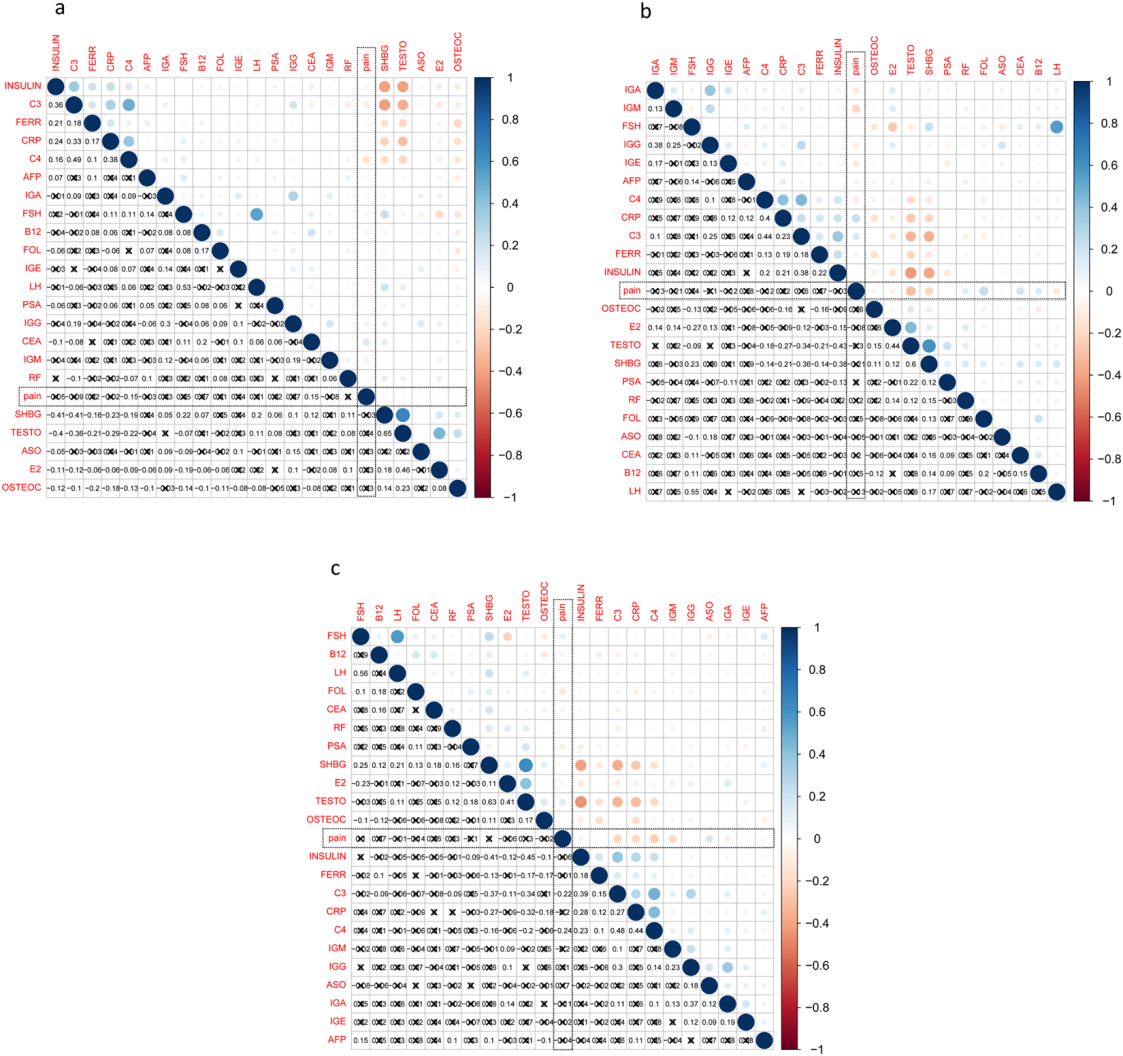
Correlation analysis between pain and 22 biochemical markers with Pearson. *(**a**) CPPS vs Non-CPPS; (**b**) IIIa vs Normal; (**c**) IIIb vs Normal. *The Pearson coefficients were shown in the left lower triangle. *Cross symbols indicated the associations were insignificant.

In order to investigate the associations between prostatitis and the biochemical markers investigated, correlation analyses were conducted for CPPS vs non-CPPS, IIIa vs normal, and IIIb vs normal sub-groups. The results suggested that pain was significantly associated with CEA (*r* ^2^ = 0.153, *p* = 0.032) and C4 (*r* ^2^ = −0.154, *p* = 0.031) in the CP/CPPS vs non-CPPS analysis (Fig. 1a). While no association was evident in the IIIa vs normal analysis (Fig. 1b), negative correlations were observed for C3 (*r* ^2^ = −0.220, *p* = 0.042) and C4 (*r* ^2^ = −0.240, *p* = 0.026) in the IIIb vs normal analysis (Fig. 1c). These associations suggest that there may be some relationships between CP/CPPS, inflammatory/immune factors, and tumor-related proteins. Further analyses were necessary.

### Inflammatory/immune markers, hormone markers, and tumor-related proteins are significantly associated with CP/CPPS

In the first step of analysis, the participants were divided into CP/CPPS (pain ≥ 4) and non-CPPS (pain = 0) groups. However, in the baseline analyses, no significant correlations were observed (Table 1). In order to investigate further, regression analyses were performed. In the logistic regression analyses, the participant category (CP/CPPS or non-CPPS) was treated as the dependent variable. Osteocalcin was the only statistically significant predictor in the unadjusted model (OR = 0.610, 95% CI = 0.379–0.983, *p* = 0.042) (Table S1). Furthermore, CEA (unadjusted: B = 0.087, 95% CI = 0.008–0.166, *p* = 0.032) and C4 (unadjusted: B = −0.179, 95% CI = −0.341–0.017, *p* = 0.031; age-adjusted: B = −0.187, 95% CI = −0.349–0.025, *p* = 0.024; age and smoking-adjusted: B = −0.195, 95% CI = −0.361–0.028, *p* = 0.022; multivariate-adjusted: B = −0.231, 95% CI = −0.406–0.056, *p* = 0.010) were also identified to be significant predictors of CP/CPPS after linear regression analysis (Table S2).

**Table 1:** The baseline characteristics of participants in our analysis.

		CPPS (Pain ≥ 4)	Non-CPPS (Pain = 0)	P^a^
NO.		198	1241	
Age		37.25 ± 10.08	35.80 ± 10.35	0.067
BMI		23.28 ± 3.39	23.27 ± 3.36	0.948
WHR		0.88 ± 0.06	0.88 ± 0.06	0.544
Smoke	Y	107	651	
	N	85	562	
Drink	Y	172	1073	
	N	24	159	0.797
AFP		2.56 ± 1.55	2.56 ± 1.45	0.719
B12		699.96 ± 237.03	682.04 ± 230.22	0.334
CEA		2.42 ± 1.57	2.46 ± 1.60	0.766
E2		34.29 ± 9.36	34.63 ± 9.99	0.854
FERR		386.59 ± 238.00	370.39 ± 224.53	0.544
FOL		9.75 ± 2.73	9.52 ± 2.79	0.203
FSH		6.43 ± 5.99	5.85 ± 4.12	0.211
IgE		268.24 ± 434.27	285.14 ± 500.23	0.431
Insulin		8.44 ± 9.93	8.28 ± 8.25	0.449
LH		5.72 ± 3.11	5.48 ± 2.38	0.460
Osteoc		23.98 ± 8.41	25.25 ± 8.57	0.042
SHBG		41.49 ± 20.93	40.85 ± 20.37	0.683
TESTO		6.20 ± 1.91	6.31 ± 1.91	0.314
PSA		1.03 ± 1.02	0.96 ± 0.86	0.426
CRP		1.57 ± 4.62	1.33 ± 2.73	0.246
IgG		13.28 ± 2.49	13.40 ± 2.58	0.700
IgA		2.50 ± 0.97	2.50 ± 0.90	0.828
IgM		1.39 ± 0.72	1.45 ± 0.79	0.395
C3		1.13 ± 0.24	1.12 ± 0.22	0.728
C4		0.33 ± 0.10	0.33 ± 0.09	0.755
ASO		77.12 ± 73.46	84.68 ± 80.04	0.136
RF		5.92 ± 6.69	6.85 ± 8.73	0.633

*The CP/CPPS was defined with pain evaluated by National Institutes of Health Chronic Prostatitis Symptom Index (NIH-CPSI). *CPPS: pain ≥ 4; Non-CPPS: pain = 0. *^a^the p values were calculated with Student’s t test, Mann—Whitney U test, and the Χ2 test. *BMI = Body Mass Index; WHR = Waist Hip Rate; C3 = Complement 3; AFP = Alpha-fetoprotein; B12 = Vitamin B12; CEA = Carcino Embryonie Antigen; E2 = Estradiol; FERR = Ferritin; FOL = Folic acid; FSH = Follicle-Stimulating Hormone; IgE = Immunoglobulin E; LH = Luteinizing Hormone; Osteoc = Osteocalcin; SHBG = Sex Hormone Binding Globulin; TESTO = Testosterone; PSA = Prostate Specific Antigen; CRP = C reactive protein; ASO = Aso antistrptolysin o; RF = Rheumatoid Factor.

Next, the levels of all the biochemical markers were divided into quartiles. Multinomial logistic regression was conducted, with the lowest quartile as the reference. The results suggested that C3 (Q2; Model 1: OR = 0.530, 95% CI = 0.338–0.832, *p* = 0.006; Model 2: OR = 0.535, 95% CI = 0.341–0.840, *p* = 0.007; Model 3: OR = 0.513, 95% CI = 0.324–0.813, *p* = 0.004; Model 4: OR = 0.504, 95% CI = 0.316–0.804, *p* = 0.004), testosterone (Q2; Model 1: OR = 0.531, 95% CI = 0.338–0.834, *p* = 0.006; Model 2: OR = 0.535, 95% CI = 0.341–0.841, *p* = 0.007; Model 3: OR = 0.518, 95% CI = 0.328–0.818, *p* = 0.005; Model 4: OR = 0.522, 95% CI = 0.326–0.834, *p* = 0.007), and CRP (Q3; Model 1: OR = 0.605, 95% CI = 0.382–0.956, *p* = 0.032; Model 2: OR = 0.548, 95% CI = 0.344–0.875, *p* = 0.012; Model 3: OR = 0.521, 95% CI = 0.322–0.844, *p* = 0.008; Model 4: OR = 0.503, 95% CI = 0.303–0.834, *p* = 0.008) were associated with CP/CPPS (Table S3).

We next performed ROC analysis by combining the variables (Osteoc, CEA, C4, C3, TESTO, and CRP) with significant association in regression analyses. Although this model combined all the significant variables with essential participant information, the predictive function was not satisfactory (AUC = 0.583, 95% CI = 0.539–0.626, *p* = 0.001). On the other hand, this finding confirms the complexity of CP/CPPS, which could not be explained by the limited biochemical markers that we investigated (Table 2, Figure S1).

**Table 2:** The ROC analysis with the significant biochemical markers in different groups (CPPS vs Non-CPPS; IIIa vs Normal; IIIb vs Normal; IV vs Normal).

Model	Items	None			Add age			Add smoke and age			Combined all*		
		AUC	95% CI	P	AUC	95% CI	P	AUC	95% CI	P	AUC	95% CI	P
CPPS vs Non-CPPS	Osteoc	0.551	0.507–0.595	0.023	0.552	0.510–0.594	0.021	0.551	0.508–0.593	0.024	0.556	0.513–0.599	0.013
	CEA	0.516	0.472–0.560	0.485	0.544	0.501–0.587	0.051	0.547	0.503–0.591	0.037	0.548	0.504–0.592	0.032
	C4	0.496	0.451–0.540	0.845	0.543	0.499–0.586	0.059	0.543	0.499–0.587	0.058	0.544	0.500–0.588	0.052
	Osteoc, CEA, C4	0.548	0.504–0.593	0.032	0.552	0.509–0.596	0.020	0.552	0.508–0.595	0.021	0.555	0.510–0.599	0.015
	Osteoc, CEA, C4, C3^a^, Testo^a^, CRP^a^	0.569	0.524–0.615	0.004	0.571	0.527–0.615	0.003	0.572	0.528–0.616	0.003	0.583	0.539–0.626	0.001
IIIa vs Normal	FERR	0.639	0.534–0.745	0.006	0.642	0.535–0.750	0.005	0.644	0.537–0.751	0.005	0.652	0.550–0.753	0.003
	FSH	0.574	0.479–0.668	0.147	0.591	0.497–0.684	0.075	0.592	0.498–0.686	0.071	0.631	0.529–0.732	0.010
	FERR, FSH	0.637	0.529–0.745	0.007	0.638	0.531–0.746	0.007	0.637	0.529–0.745	0.007	0.644	0.539–0.749	0.005
	FERR, FSH, Insulin^a^	0.639	0.530–0.747	0.006	0.639	0.531–0.747	0.006	0.637	0.529–0.746	0.007	0.664	0.560–0.768	0.001
IIIb vs Normal	IGE	0.556	0.489–0.624	0.099	0.556	0.488–0.624	0.104	0.561	0.492–0.629	0.076	0.597	0.532–0.661	0.005
	ASO	0.573	0.508–0.638	0.032	0.570	0.504–0.636	0.042	0.570	0.505–0.636	0.040	0.593	0.527–0.658	0.007
	CRP	0.565	0.491–0.640	0.071	0.578	0.504–0.651	0.032	0.567	0.495–0.640	0.062	0.577	0.507–0.647	0.033
	C3	0.509	0.441–0.577	0.796	0.526	0.463–0.589	0.453	0.530	0.464–0.596	0.381	0.559	0.494–0.624	0.086
	C4	0.483	0.414–0.551	0.610	0.530	0.465–0.594	0.385	0.532	0.468–0.597	0.348	0.559	0.494–0.623	0.087
	IgE, ASO, CRP, C3, C4	0.589	0.519–0.659	0.014	0.593	0.522–0.663	0.010	0.594	0.524–0.664	0.009	0.619	0.551–0.688	0.001
	IgE, ASO, CRP, C3, C4, RF^a^, PSA^a^, Osteoc^a^	0.636	0.567–0.705	< 0.001	0.638	0.570–0.707	< 0.001	0.639	0.570–0.707	< 0.001	0.676	0.608–0.743	< 0.001
IV vs Normal	Osteoc	0.570	0.515–0.624	0.014	0.628	0.574–0.681	< 0.001	0.638	0.586–0.691	< 0.001	0.639	0.586–0.692	< 0.001
	FSH	0.552	0.496–0.609	0.065	0.625	0.573–0.679	< 0.001	0.635	0.583–0.687	< 0.001	0.633	0.581–0.686	< 0.001
	PSA	0.605	0.552–0.658	< 0.001	0.653	0.603–0.704	< 0.001	0.659	0.609–0.709	< 0.001	0.653	0.601–0.705	< 0.001
	Osteoc, FSH, PSA	0.629	0.577–0.681	< 0.001	0.653	0.601–0.704	< 0.001	0.660	0.609–0.710	< 0.001	0.658	0.607–0.710	< 0.001

*All the factors added in analysis included age, smoke, drink, BMI and WHR. ^a^The biochemical markers were discovered in the multivariate logistic regression analysis.

### New factors discovered in association with NIH-IIIa prostatitis

The analysis of the CP/CPPS vs non-CPPS group revealed the factors that influence CP/CPPS. However, as a complex disease, CP/CPPS can also be divided into two sub-groups (NIH-IIIa and NIH-IIIb), and non-CPPS (NIH-IV and normal); thus it may have potential associations among the various sub-groups. To investigate this, the IIIa vs normal group was investigated in further analyses. As expected, new biochemical markers were identified in the baseline analysis for this group (FERR: *p* = 0.015; FSH: *p* = 0.049) (Table 3). In logistic regression analyses, FERR and FSH (unadjusted: OR = 1.813, 95% CI = 1.000–3.287, *p* = 0.050) were again found to be risk factors. This was particularly evident for FERR, which was statistically significant even after multivariate-adjustment (unadjusted: OR = 2.273, 95% CI = 1.190–4.341, *p* = 0.013; age-adjusted: OR = 2.131, 95% CI = 1.125–4.034, *p* = 0.020; age and smoking-adjusted: OR = 2.156, 95% CI = 1.128–4.120, *p* = 0.020; multivariate-adjusted: OR = 2.003, 95% CI = 1.021–3.932, *p* = 0.043) (Table S4). However, no new biochemical markers were discovered in the linear regression analyses (Table S5).

**Table 3:** The baseline features of IIIa, IIIb, IV and Normal controls on the basis of National Institute of Diabetes and Digestive and Kidney Diseases (NIDDK) classification system.

		Pain ≥ 4				Pain = 0			
		IIIa	P^a^	IIIb	P^b^	IV	P^c^	Normal	P^d^
NO.		35		86		139		416	
Age		37.29 ± 9.93	0.104	35.33 ± 8.82	0.496	38.84 ± 10.19	** < 0.001**	34.57 ± 9.43	** < 0.001**
BMI		24.33 ± 3.49	0.102	22.99 ± 3.21	0.332	34.57 ± 9.43	0.290	23.37 ± 3.30	0.151
WHR		0.90 ± 0.05	0.078	0.87 ± 0.06	0.374	0.89 ± 0.05	0.037	0.88 ± 0.05	0.036
Smoke	Y	17		39		77		198	
	N	18	0.912	47	0.704	62	0.111	218	
Drink	Y	28		78		115		366	
	N	7	0.184	8	0.473	24	0.115	50	
AFP		2.53 ± 1.89	0.631	2.52 ± 1.31	0.831	2.53 ± 1.25	0.605	2.51 ± 1.54	0.895
B12		740.62 ± 247.24	0.079	715.49 ± 248.97	0.124	693.49 ± 253.50	0.352	668.63 ± 226.87	0.167
CEA		2.59 ± 1.70	0.602	2.29 ± 1.48	0.987	2.44 ± 1.48	0.395	2.37 ± 1.56	0.809
E2		31.53 ± 9.68	0.099	33.29 ± 8.60	0.583	34.36 ± 9.78	0.919	34.35 ± 9.96	0.361
FERR		479.67 ± 269.11	0.015	354.24 ± 201.10	0.633	392.40 ± 234.81	0.234	359.59 ± 207.34	0.065
FOL		9.36 ± 2.58	0.881	9.86 ± 2.72	0.230	9.76 ± 2.60	0.241	9.54 ± 2.97	0.455
**FSH**		7.57 ± 8.85	**0.049**	5.84 ± 5.00	0.839	6.32 ± 3.98	**0.037**	5.62 ± 4.05	0.067
IgE		247.16 ± 369.75	0.793	214.12 ± 284.53	0.060	248.00 ± 322.96	0.387	286.84 ± 418.73	0.292
Insulin		8.55 ± 5.90	0.640	7.44 ± 5.94	0.176	8.10 ± 5.51	0.912	8.76 ± 9.81	0.544
LH		5.94 ± 3.41	0.448	5.59 ± 2.94	0.857	5.73 ± 2.58	0.363	5.41 ± 2.24	0.759
**OSTEOC**		23.55 ± 6.48	0.211	24.60 ± 8.60	0.253	23.31 ± 6.78	**0.006**	25.46 ± 8.64	**0.040**
SHBG		35.57 ± 12.12	0.289	41.40 ± 18.57	0.506	41.06 ± 20.81	0.501	40.35 ± 21.19	0.514
TESTO		5.76 ± 1.95	0.091	6.12 ± 1.81	0.520	6.15 ± 1.83	0.659	6.26 ± 1.90	0.366
**PSA**		1.17 ± 1.21	0.155	0.98 ± 1.00	0.163	1.20 ± 1.38	** < 0.001**	0.90 ± 0.80	**0.001**
CRP		2.50 ± 7.83	0.274	1.73 ± 4.81	0.205	1.77 ± 4.50	0.449	1.22 ± 2.30	0.276
IgG		13.25 ± 2.56	0.973	13.33 ± 2.17	0.657	13.37 ± 2.64	0.678	13.25 ± 2.43	0.956
IgA		2.34 ± 0.84	0.393	2.47 ± 0.85	0.950	2.61 ± 0.92	0.223	2.49 ± 0.92	0.453
IgM		1.43 ± 0.84	0.466	1.42 ± 0.75	0.370	1.54 ± 0.89	0.876	1.51 ± 0.86	0.714
C3		1.16 ± 0.22	0.376	1.13 ± 0.26	0.820	1.13 ± 0.21	0.628	1.12 ± 0.23	0.800
C4		0.35 ± 0.10	0.322	0.33 ± 0.11	0.982	0.33 ± 0.09	0.829	0.33 ± 0.09	0.782
ASO		87.29 ± 86.04	0.725	74.84 ± 85.44	0.040	89.60 ± 88.61	0.833	87.11 ± 80.24	0.248
RF		5.14 ± 5.92	0.828	5.70 ± 6.29	0.557	6.47 ± 8.37	0.918	6.30 ± 7.61	0.939

*^a^The T test between IIIa and Normal group; ^b^The T test between IIIb and Normal group; ^c^The T test between IV and Normal group; ^d^One-Way ANOVA.

In the quartered analyses, FERR was confirmed to be a risk factor. With an increasing level of FERR, there was a greater risk of NIH-IIIa prostatitis (Q4; Model 1: OR = 3.128, 95% CI = 1.185–8.259, *p* = 0.021; Model 2: OR = 3.041, 95% CI = 1.149–8.049, *p* = 0.025; Model 3: OR = 3.100, 95% CI = 1.163–8.266, *p* = 0.024; Model 4: OR = 2.837, 95% CI = 1.019–7.898, *p* = 0.046; *p* for trend = 0.010). In addition, insulin was unexpectedly identified as a significant predictor (Q2; Model 1: OR = 0.184, 95% CI = 0.039–0.859, *p* = 0.031; Model 2: OR = 0.188, 95% CI = 0.040–0.881, *p* = 0.034; Model 3: OR = 0.188, 95% CI = 0.040–0.882, *p* = 0.034; Model 4: OR = 0.171, 95% CI = 0.036–0.815, *p* = 0.027) (Table S6).

**Figure 2:**
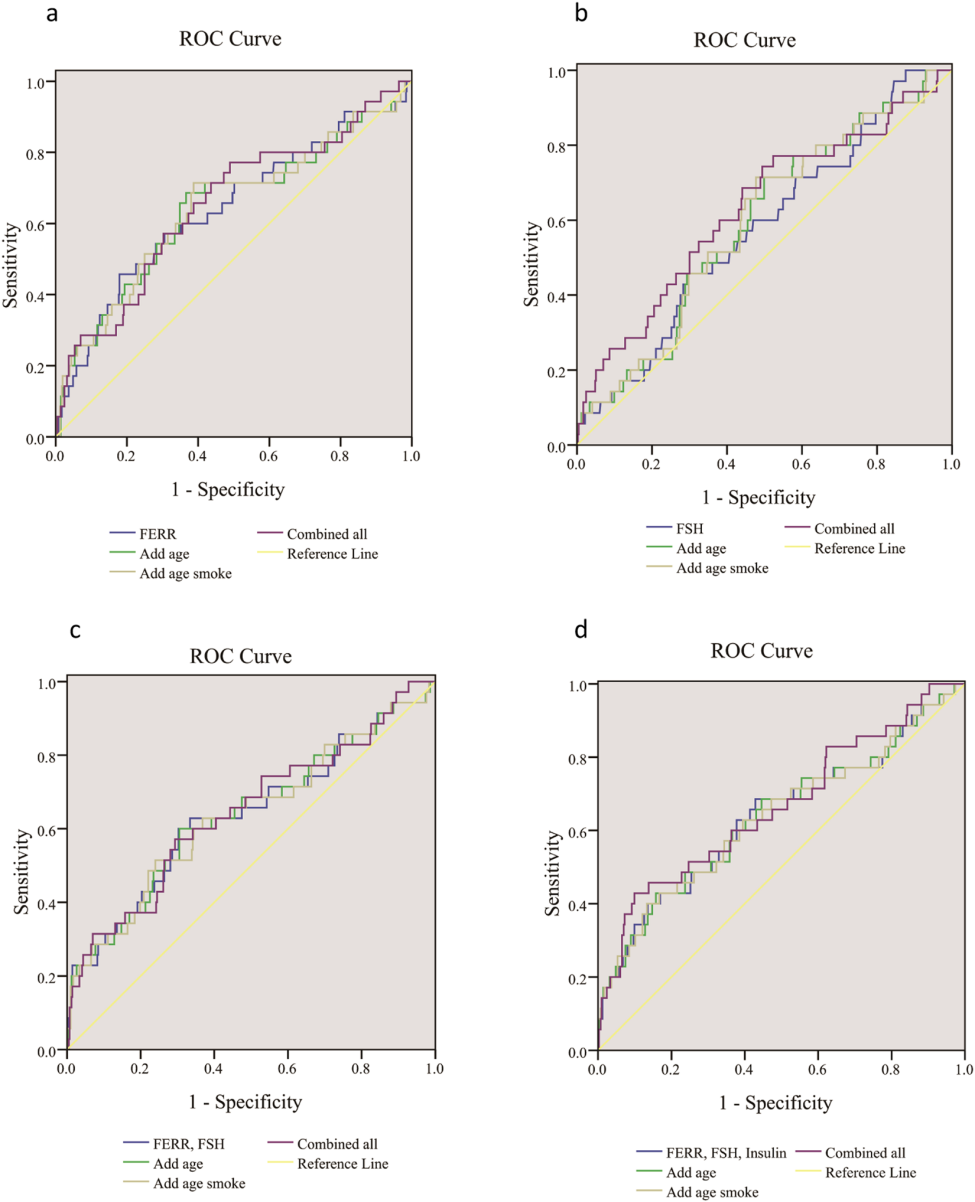
The ROC analysis with the significant biochemical markers for IIIa vs Normal group. *(**a**) FERR; (**b**) FSH; (**c**) FERR and FSH; (**d**) add biochemical markers discovered in multivariate logistic regression analysis.

The results of ROC analysis suggested that FERR was a stronger predictor (AUC = 0.639, 95% CI = 0.534–0.745, *p* = 0.006) than any other independent predictor. Even with the addition of other factors (age, smoking, alcohol consumption, BMI, WHR, FSH, and insulin), the AUC only increased slightly to 0.664 (*p* = 0.001) (Table 2, Fig. 2).

### Inflammatory/immune factors, PSA, and osteocalcin are important in NIH-IIIb prostatitis

For the IIIb vs normal group analyses, no significant associations were identified in the baseline analyses (Table 3). In the regression analyses, several Inflammatory/immune markers (C3, C4, and CRP) were consistently found to be significant. In addition, two new biochemical markers were also identified: IgE (multivariate-adjusted: OR = 0.829, 95% CI = 0.691–0.994, *p* = 0.043) and ASO (un-adjusted: OR = 0.763, 95% CI = 0.588–0.989, *p* = 0.041; age-adjusted: OR = 0.765, 95% CI = 0.590–0.993, *p* = 0.044; age and smoking-adjusted: OR = 0.767, 95% CI = 0.591–0.997, *p* = 0.047) (Tables S7–S9). In the following quartered analyses, osteocalcin (Q3; Model 1: OR = 0.504, 95% CI = 0.257–0.987, *p* = 0.046; Model 2: OR = 0.512, 95% CI = 0.260–1.009, *p* = 0.053; Model 3: OR = 0.506, 95% CI = 0.256–0.999, *p* = 0.050; Model 4: OR = 0.454, 95% CI = 0.226–0.913, *p* = 0.027), RF (Q2; Model 1: OR = 2.441, 95% CI = 1.178–5.058, *p* = 0.016; Model 2: OR = 2.469, 95% CI = 1.190–5.122, *p* = 0.015; Model 3: OR = 2.449, 95%CI = 1.179–5.088, *p* = 0.016; Model 4: OR = 2.394, 95% CI = 1.148–4.991, *p* = 0.020), and PSA (Q3; Model 1: OR = 2.346, 95% CI = 1.173–4.693, *p* = 0.016; Model 2: OR = 2.325, 95% CI = 1.162–4.656, *p* = 0.017; Model 3: OR = 2.314, 95% CI = 1.155–4.639, *p* = 0.018; Model 4: OR = 2.279, 95% CI = 1.130–4.596, *p* = 0.021) were also found to be associated with the development of NIH-IIIb prostatitis (Table S9).

In addition, when combining all the biochemical markers (IgE, ASO, CRP, C3, and C4) and confounding factors (age, smoking, alcohol consumption, BMI, and WHR) identified in the regression analyses, the ROC AUC reached 0.619 (*p* = 0.001). By adding RF, PSA, and osteocalcin to the model, the AUC significantly increased to 0.676 (*p* < 0.001) (Table 2, Figure S2).

### Osteocalcin, FSH, and PSA are also associated with NIH-IV prostatitis

With regard to type IV prostatitis, osteocalcin (*p* = 0.006), FSH (*p* = 0.037), and PSA (*p* < 0.001) showed significant difference in the IV vs normal group baseline analyses (Table 3). We next performed logistic regression analysis in the sub-group without pain. This analysis confirmed the association of osteocalcin (un-adjusted: OR = 0.402, 95% CI = 0.208–0.779, *p* = 0.007), FSH (un-adjusted: OR = 1.447, 95% CI = 1.020–2.051, *p* = 0.038), and PSA (un-adjusted: OR = 1.882, 95% CI = 1.358–2.609, *p* < 0.001; age-adjusted: OR = 1.720, 95% CI = 1.234–2.397, *p* < 0.001; age and smoking-adjusted: OR = 1.698, 95% CI = 1.218–2.368, *p* = 0.002; multivariate-adjusted: OR = 1.731, 95% CI = 1.234–2.429, *p* = 0.001) with type IV prostatitis (Table S10). In the subsequent quartered analysis, PSA and osteocalcin were found again to be statistically significant predictors. In addition, it appeared that, with increased levels of PSA (*p* for trend = 0.001) and osteocalcin (*p* for trend = 0.010), the risk and protective effects were enhanced, respectively (Table S11). The ROC analysis further indicated that these three biochemical markers had a potential predictive value for NIH-IV prostatitis (AUC = 0.658, 95% CI = 0.607–0.710, *p* < 0.001) (Table 2, Figure S3).

### Discriminant and principal component analyses

Next, we performed discriminant analysis, and found that osteocalcin was a statistically significant predictor for CPPS, with a false positive rate (FPR) = 45.59%. For NIH-IV prostatitis, osteocalcin and PSA were identified as predictors, with a lower FPR (original: 39.10%; cross-validated: 39.64%). Unexpectedly, only FERR was confirmed to be significantly associated with NIH-IIIa prostatitis, with the lowest FPR (38.36%) (Table 4). These results suggest that PSA, osteocalcin, and FERR are most probably vital factors in the development of CP/CPPS and other types of prostatitis. However, with the inclusion of all the significant biochemical markers for each group, CP/CPPS, NIH-IIIa, NIH-IIIb, and NIH-IV types of prostatitis could not be distinguished from each other (Figure S4).

**Table 4:** The results of discriminant analysis for CPPS vs Non-CPPS, IIIa vs Normal, IIIb vs Normal and IV vs Normal groups.

	Discriminant factor	Wilk’s Lambda	P		PGM (Original)			PGM (Cross-validated)		
					Non-CPPS	CPPS	FPR	Non-CPPS	CPPS	FPR
CPPS vs Non-CPPS	Osteoc	0.994	0.013	Non-CPPS	675 (54.4%)	566 (45.6%)		675 (54.4%)	566 (45.6%)	
				CPPS	90 (45.5%)	108 (54.5%)	45.59%	90 (45.5%)	108 (54.5%)	45.59%
IIIa vs Normal	FERR	0.973	0.002	Non-CPPS	257 (61.8%)	159 (38.2%)		257 (61.8%)	159 (38.2%)	
				CPPS	14 (40.0%)	21 (60.0%)	38.36%	14 (40.0%)	21 (60.0%)	38.36%
IIIb vs Normal	NA	NA	NA	NA	NA	NA	NA	NA	NA	NA
IV vs Normal	PSA	0.984	0.010	Non-CPPS	257 (61.8%)	159 (38.2%)		254 (61.1%)	162 (38.9%)	
	Osteoc	0.973	0.003	CPPS	58 (41.7%)	81 (58.3%)	39.10%	58 (41.7%)	81 (58.3%)	39.64%

*FPR = false positive rate; PGM = Predicted Group Membership. *22 biochemical markers in our study were included in this analysis.

## Discussion

Chronic prostatitis is a complex syndrome diagnosed worldwide. Despite its high morbidity and impact on the health of numerous men, little is known about the environmental and inherited risk factors to the pathogenesis of prostatitis. In order to define the disease severity, the NIH-CPSI was developed. On the basis of this system, our study was performed to uncover potential risk factors in association with CP/CPPS and NIH-IV prostatitis, and to evaluate their predictive effects for the disease. After the comprehensive analysis, we discovered several types of associations (CPPS vs non-CPPS: Osteoc, CEA, C4, C3, TESTO, and CRP; IIIa vs normal: FERR, FSH, and insulin; IIIb vs normal: IgE, ASO, CRP, C3, C4, RF, PSA, and Osteoc; IV vs normal: Osteoc, FSH, and PSA), in which osteocalcin was reproducibly found to be a protective factor in three groups, including CP/CPPS, NIH-IIIb, and NIH-IV prostatitis. Despite the fact that various predictors were discovered for the different groups, our further discriminant analysis only revealed FERR and PSA in association with NIH-IIIa and NIH-IV prostatitis, respectively.

Osteocalcin contains a 46–50 amino acid residues and is a molecular weight of 5.6 kDa secreted protein, which is produced primarily by osteoblasts^34^. Osteocalcin was initially isolated by Price *et al*.^35, 36^ from bovine and human bone. In human, osteocalcin is encoded by the *BGLAP* gene, locating at 1q25–q31, and encoding an 11 kD pre-pro-protein with 98 amino acid residues^37^. Some early studies mainly focused on its function in bone growth^38^. However, recent reports showed that this protein has extensive functions, not limited in bone. In 2007, Lee *et al*.^39^ revealed endocrine effects of osteocalcin, probably playing roles in glucose metabolism. Ferron *et al*.^40^ further confirmed this function and indicated that osteocalcin might be valuable in the treatment of metabolic diseases. In addition, osteocalcin may function in Leydig cells of the testis to stimulate testosterone biosynthesis, and therefore affect male fertility^41^. The mechanism might be related to the pancreas-bone-testis axis^42^. Although there is no direct evidence of an association of osteocalcin with CP/CPPS and type IV prostatitis, researchers observed that osteocalcin may play a role in brain function. In 2013, Oury *et al*.^43^ revealed that, compared to control, the cognition of osteocalcin null mice was impaired, with anxiety and depression-related behavior. These manifestations were similar, to some extent, as those observed in CP/CPPS^44^. In addition, Woodworth *et al*.^45^ identified unique microstructural changes in the brain of urological chronic pelvic pain syndrome (UCPPS). These results suggested that osteocalcin might be a protective factor, which is consistent with our results, especially with regard to the psychological aspect. The possible mechanism might be that osteocalcin functions as a neuropeptide in the brain of CP/CPPS (NIH-III prostatitis) patients^46^. On the other hand, NIH-IV prostatitis is asymptomatic with persistent leukocytosis. Unlike NIH-III prostatitis, recent studies suggested that the inflammatory process may play a potential role in the pathogenesis^47, 48^. Moreover, our previous studies identified that osteocalcin might be a protective factor for low-grade inflammation^49, 50^. Therefore, it seems reasonable that osteocalcin could influence the inflammatory process of NIH-IV prostatitis.

PSA is also known as gamma-seminoprotein or kallikrein-3 (KLK3). As member of the kallikrein-related peptidase family, PSA is mainly secreted by the epithelial cells of the prostate gland. Its main functions are believed to be liquefying semen and dissolving cervical mucus^51^. In the serum of men, small quantities are present. Nowadays it is widely applied in the diagnosis of prostate cancer and other types of prostate diseases^52^, including the prostatitis. In 2011, Korrovits *et al*.^48^ found that in type IV prostatitis, both IL-6 in seminal plasma and PSA in blood serum were significantly increased. Similarly, Gui-Zhong *et al*.^53^ discovered an elevation of serum PSA in asymptomatic prostatitis, thus helping to prevent unnecessary repeated biopsies. On the other hand, there may be a correlation between PSA level and prostatitis to some extent. In the study by Ponniah *et al*.^22^, an autoimmune component was identified to contribute to symptoms in some men. In a follow-up study, they confirmed this finding and recommended that PSA can be the candidate antigen for further targeted therapy in CP/CPPS^23^. In our study, we validated the assertion that PSA might be a risk factor for CP/CPPS and asymptomatic type IV prostatitis. ROC analysis also showed that PSA level may be one of the elements predicting CP/CPPS. However, some studies did not observe this correlation. For examples, Engelhardt *et al*.^47, 54, 55^ focused on asymptomatic type IV prostatitis and benign prostatic hyperplasia (BPH) and did not observe any association between NIH-IV prostatitis and PSA level. In our study, the evaluation and classification of chronic prostatitis was based on the NIH-CPSI Score and EPS, whereas Engelhardt *et al*. used histopathology for evaluation and classification, which may have not strictly excluded NIH-II prostatitis patients. Furthermore, PSA has been identified to be elevated upon inflammation^56^. Therefore, we could not ignore the influence of PSA level on NIH-II prostatitis. Future studies are needed to examine more strategies to diagnose prostatitis, especially for NIH-IV, and to investigate the role of PSA in NIH-IV prostatitis.

Much to our surprise, FERR was found to be associated with NIH-IIIa prostatitis, and had the highest predictive function, compared to the other independent biochemical markers (all predictors combined with FERR: AUC = 0.652, 95% CI = 0.550–0.753, *p* = 0.003). In addition, in the discriminant analysis, FERR was shown to have lower FPR. Further, the finding indicated that the risk of NIH-IIIa prostatitis was elevated with increasing level of FERR. Although there was no direct evidence in the literature to suggest an association between FERR and prostatitis, some studies have hypothesized a potential correlation. Ferritin is a 24-subunit globular protein composed of two types of subunits (H and L). The ratio of H to L subunits varies according to the tissue type and developmental stage^57^. This protein is a universal intracellular protein, which is produced by almost all living organisms. Its main function is storing and releasing iron in a controlled fashion. Previous studies have revealed that elevated levels of serum ferritin also occurr in patients with hematologic malignancies, such as Hodgkin’s disease and acute leukemia, accompanied by impaired cell-mediated immunity^58, 59^. This suggests that ferritin might be associated to some extent with the immune system^60^. Additionally, excess iron can induce oxidative stress and inflammation^61^. As an ubiquitous intracellular protein that can store and release iron, serum ferritin was also identified to be associated with a wide range of inflammatory conditions^62^, such as renal disease^63^, autoimmune disease^64^, and infection. NIH-IIIa prostatitis is characterized by pain and obvious leukocytes in EPS, indicating that an inflammation/immune reaction is occurring. On the basis of this observation, we assumed that serum ferritin would be significantly increased in the type IIIa prostatitis.

In addition to osteocalcin, FERR, and PSA, other inflammatory/immune factors have been identified as the predictive factors. Autoimmunity might be the etiology of CP/CPPS as reported in previous studies^22, 65^. Based on this, Rudick *et al*.^66^ developed an experimental autoimmune prostatitis. In their study, prostate glands from rats were collected to inject into mice. Following this, persistent pelvic pain and inflammatory infiltrates in prostate lobes were found. In addition, neuronal fiber distribution was also significantly increased. These results confirmed the role of autoimmune factors in the development of CP/CPPS in an animal model. In our study, several significant biochemical markers were also observed in the regression analyses, including IgE, C3, C4, CRP, ASO, and RF. As important aspects of the immune response, the association between CP/CPPS and these factors seemed to be reasonable. However, these inflammatory/immune factors were mainly associated with NIH-IIIb prostatitis, rather than NIH-IIIa and NIH-IV. Based on previous studies, and the known function of these biochemical markers, we speculate that IgE, C3, C4, CRP, ASO, and RF might contribute to the pain experienced in CP/CPPS. On the other hand, Osteoc could influence the psychological symptoms of CP/CPPS. Additionally, hormonal elements (Osteoc, TESTO, FSH, and insulin), tumor-related proteins (CEA and PSA), and nutrition markers (FERR) might take part in the systematic low-grade inflammation recruiting the leukocytes to EPS. Further studies with larger sample size, with molecular experiments are needed.

Current study primarily investigated the function of inflammatory/immune markers, hormone markers, tumor-related proteins, and nutrition markers in the development of CP/CPPS (NIH-III prostatitis) and NIH-IV prostatitis. Twenty-two specific biochemical markers, covering the common biochemical markers checked in the hospital, were examined. However, some other more significant factors, such as interleukin, TNF-Alpha, etc. were not investigated. Further, in process of screening samples, strict exclusion criteria were applied. To some extent, the eligible participants included in this current study may not fully represent CP/CPPS and NIH-IV prostatitis patients. Therefore, there might have some biases in the association between the investigated biochemical markers and prostatitis. Considering these issues, we suggest that future studies could investigate more cytokines among definite non-bacterial prostatitis patients (evaluated with NIH-CPSI score, EPS, urine cultures, ejaculatory cultures, and histology for classification).

### Limitations

As discussed above, some limitations of this study need to be pointed out: (I) only 22 common biochemical markers were investigated in our *FAMHES* project. These markers covered inflammatory/immune markers, hormone markers, tumor-related proteins, and nutrition markers. Some potentially more important factors, such as interleukin, TNF-Alpha, etc. were not investigated; (II) strict exclusion criteria were applied in screening the sample, which excluded some confounding factors that could have influenced the association analyses. On the other hand, these eligible participants may not represent the real population of patients; (III) in this study, chronic prostatitis was evaluated and classified by the NIH-CPSI score and EPS only, which could result in a classification bias, and may have influenced the association between PSA and prostatitis; (IV) statistical power was not assessed in this study; (V) some significant biochemical markers were observed only in one of the adjusted models. In order to confirm these findings, analysis of larger sample size is needed, together with further molecular experiments; (VI) only five common confounding factors (age, smoking, alcohol consumption, BMI, and WHR) were included, which would influence the results of ROC analysis and observed associations.

## Conclusion

CP is a complex male urologic disease. In order to investigate the potentially associated risk factors, and evaluate their predictive value, 22 different biochemical markers were investigated, covering inflammatory/immune markers, hormone markers, tumor-related proteins, and nutrition markers. Our results indicated that inflammatory/immune factors (IgE, C3, C4, CRP, ASO, and RF), hormone elements (Osteoc, TESTO, FSH, and insulin), tumor-related proteins (CEA and PSA), and a nutrition marker (FERR) were significantly associated with CP/CPPS or one of its sub-types. Among them, osteocalcin was consistently found to be a protective factor for CP/CPPS, NIH-IIIb, and NIH-IV prostatitis. Additionally, ferritin was more valuable in predicting NIH-IIIa prostatitis, independently. Further molecular and epidemiological studies with larger sample size are needed.
